# Dr. T. F. Allen’s “High Potency Allopathy”

**Published:** 1890-06

**Authors:** B. Fincke

**Affiliations:** Brooklyn


### DR. T. F. ALLEN'S "HIGH POTENCY ALLOPATHY."

B. Fincke, M. D., Brooklyn.

"Thou shalt not bear false witness against thy neighbor."

In a letter to the editors of The Homœopathic Physician, published in the May number of this journal, the following statements are made :

Page 206. "I witness the most exclusive practitioners of our art reporting cures with the highest potencies of a drug which has never been proved, whose indications are wholly clinical. This surely is not Homœopathy. It is certainly high potency allopathy. There is, I am sorry to see, an increasing tendency, even among those who use the highest potencies, to prescribe from clinical indications, and to depart from the strict law of Homœopathy."

Page 205. "For twenty-five years and more I have faithfully and conscientiously practiced and taught straight Homœopathy as I understand it." But, suppose he don't understand it as Hahnemann did? Hahnemann stands upon a rock, but if the witness does not stand upon this rock but only upon his own understanding, his standpoint is a quicksand which must swallow him with all his straightforwardness. This danger is the more to be apprehended as he humbly professes his ignorance after twenty-five years' study of materia medica. Now those "exclusive practitioners" *are* standing upon that rock of Hahnemann. They are the thorough homœopathicians of Hahnemann. They accept and verify in their practice his teachings uncompromisingly when he demands that the homœopathician should heal by simple similar remedies proved upon the healthy, in the least possible dose, or a high potency, a term which was introduced by him, as can be seen in his writings in numerous places. How can the witness call these disciples of Hahnemann exclusive practitioners of his art, if he means to teach and practice straight, strict, pure Homœopathy and consequently claims the honorable name of homœopathician (Homœopathiker of Hahnemann)? If the witness is, according to his own testimony, profoundly impressed with his own ignorance after twenty-five years' study of our materia medica (page 205), why should he impute this, his own ignorance, to those exclusive practitioners who are the real homœopathicians of our art and science? We know of those who as a general rule use the high and highest potencies according to *Similia similibus* in remedy and dose, and it is a well-known general excuse of those whom the witness calls mongrels, that they are not versed enough in materia medica to prescribe accurately for a high-potency practice, a confession which implies a great compliment for those "exclusives" who not only understand how to do so, but also practice it. They certainly cannot be accused, as the witness has it, as those "who are extremely liable to neglect the study of materia medica and overdose their patients," such as indeed is the practice of mongrelism (page 205) which he abhors. But his objection is not now directly against the administration of high and highest potencies at all, which, at any rate, marks progress, because only five years ago they were "the laughing-stock of all right-minded men," but against those exclusive practitioners who in using homœopathic high potencies "practice allopathy" (page 206). Surely always the unexpected happens, and this unexpected discovery was reserved for the witness because nobody else ever thought of uniting such disjunctive ideas.

Anybody not familiar with the goings on in the homœopathic profession would think that these exclusives and high potentialists are a traitorous set of men who labor to destroy Homœopathy altogether, that they practice allopathy "*ab usu in morbis*," according to "wholly clinical indications," because they don't care a fig for provings and homœopathic materia medica pura. As such they are denounced by the witness, and thus they are held up to his admiring students and professional brethren, as the wolves in sheep's clothes, who have stolen into the fold of the professors of straight Homœopathy. Never could men be more mistaken. If these much accused, and, alas! slandered exclusives make use of clinical symptoms, it is of those which have been cured with one single remedy. These clinical symptoms, almost from the beginning of Homœopathy, have been found useful in practice and marked in the materia medica books with a degree sign (°) indicating that they are not merely clinical, but also pathogenetic symptoms, healed by such and such a remedy. They, of course, have not the value of such symptoms as have been observed upon the healthy in proving and verified in healing, but still they are useful if they form part of the totality of the symptoms, which is paramount in the selection of the remedy. But when they once shall have been verified by provings upon the healthy in the future, they will acquire the dignity of the star-symptoms.

That a remedy carefully observed—and as carefully as in a proving—to have healed a certain state of the organism in disease is to be ranged, *conversely*, as pathopoetic, and, therefore, capable of healing in a similar pathogenetic state of the organism, because it forms a feature of the pathogenetic picture does not require any further argument. And this is the point which the witness does not seem to be aware of. The clinical symptom used for healing could not be of any avail if it were not homœopathic to the case.

Besides these clinical symptoms available for cure, symptoms frequently appear after the administration of a remedy, especially when given in a high potency which do not belong to the disease under treatment, but to the remedy. These are true pathopoetic symptoms which, though observed upon the sick, can be added to those observed from this remedy upon the healthy, as Hahnemann says. (See *Organon*, § 142.)

Perhaps an example may make this clearer. In the afternoon of May 1st a heavy thunder-storm, with pouring rain, cleared and cooled the atmosphere. This may have been the cause of a sharp pain which I felt next morning after awaking in the external condyle of the humerus going down half the ulna on moving it, worse on motion and touch. At first I thought it the result of a false position during sleep, but as it would not subside and was very inconvenient, and never having had anything like it before, I took a few globules of Bryonia-alba$^{45m}$ (F.) about two P. M.

No change occurred till after midnight, when I woke up with flatulent pain in the lower abdomen, followed by a loose passage, mixed with solid lumps and the sensation of a ring half an inch thick, corresponding with the anus, with smarting and pricking as of little sticks. Another mushy stool was passed later in the night and one in the morning after rising, and several other similar passages occurred during the day, with the same sensation in ano, which I never had before. On going out I noticed a stiffness of the muscles in the right side of the neck. But the pain, for which Bryonia was taken, was almost gone, nothing remaining there but a little sensitiveness to touch, which wore off gradually.

Now, here we have, first, the healing action of the law; then, second, the pathopoetic action of Bryonia in the organs of defecation, which everybody can see to be similar to the symptoms observed upon the healthy in the materia medica; even the stiff neck is there. And, third, Bryonia might have produced other symptoms of its own not yet in the materia medica, which would have to be marked likewise as pathopoetic symptoms. But, fourth, if in my case there had been symptoms not yet observed upon the healthy, but in a cure by Bryonia (with exclusion of other remedies) alone, they would have been as well indicated as the other pathopoetic indications—for they would have been hygiopoetic symptoms—*i. e.*, obtained by healing—and all these four modifications would have the right to be called homœopathic indications.

The witness, according to his utterances, would be the last to expect from a high potency, a Cm or a M (million) centesimal, an allopathic effect, for then he would acknowledge the reality of the high potency as much as that of the low potency which he uses in his practice.

If these high potencies act at all, it is because they find their action in the organism which, changed by disease in its state, has become homœopathic to it, and hence, the artificial disease, produced by the high potency, steps into the place of the natural disease, equalizing the life-force, and health is restored according to the law of dynamics. Action and reaction are equal and directed to contrary sides, which, applied to medicine, is the homœopathic principle, *Similia similibus curantur*.

As to the other contents of that remarkable document, they speak for themselves and need no comment.

